# Not a Thing to Wear

**Published:** 1909-10

**Authors:** W. A. Philpott


For Texas Medical Journal.

### Not a Thing to Wear.

BY W. A. PHILPOTT, JR.

You have often heard it stated that when man was first created,
He adorned himself in Paradise with raiment antiquated;
And with Eve 'twas just as true, she would gather leaves a few
Then would garb herself in garments quite abbreviated, too!

Thru the summer and the autumn wardrobes were as nature wrought 'em,—
Evergreens and sheathy foliage could be found where e'er they sought 'em;
But with winter on the leaves, things were difficult; poor Eve's
Summer dresses shrank until they scarcely reached below her knees!

So 'twas during this perdition, with all leaves in seared condition,
That Eve uttered an expression that has outlived all tradition:
Once while sitting nude and bare, little head bowed in despair,
She exclaimed, "My dearest Adam, I have not *a thing* to wear!"

As to one thing we'll agree, with no leaf on any tree
Mother Eve was sore afflicted—we accept her *truthful* plea;
Ever since the creatures fair have been causing men to swear
When deceiving us by claiming *"not a blooming thing to wear!"*

Easter, 1909, April 11th.

